# Supplementary material for: A Synthetic Cytotoxic T cell Platform for Rapidly Prototyping TCR Function
Source: bioRxiv. 2023 Nov 21:2023.11.20.567960. Preprint. [Version 1] doi: 10.1101/2023.11.20.567960 (PMC10690155; doi:10.1101/2023.11.20.567960)
Supplement: Supplement 1 — Supplementary Figure 1. Intra-experiment variance of T0 controls is consistent. A summary of cell counts obtained in live targets gate of YT-rCTL/KFRET T0 controls illustrates variance between absolute counts in individual samples. While statistically significant differences were apparent between groups prepared for different experimental runs (e.g., on different days), samples prepared in the same experimental run (e.g. on the same day) are found to be highly consistent, even when comparing T0 controls composed of differing cell lines. By Levene’s test, the variance between groups of samples prepared in different batches is found to be homogenous, indicating that the use of relative cell counting as a measure of cell dropout over co-culture duration is reliable when matched experimental and T0 samples are prepared in parallel. Supplementary Figure 2. CAR-mediated cytotoxicity is primarily triggered through the CD3ζ signaling domain. K562 cells modified to express FRET2 reporter and CD19 protein coding sequence were co-incubated with YT-Indy modified with either an FMC63–41BB-CD3ζ anti-CD19 CAR or a prematurely truncated FMC63–41BB anti-CD19 CAR (CD3ζneg) at 1:1 effector:target ratios for 4 hours. Supplementary Figure 3. Lentiviral vector uptake is three orders of magnitude more efficient in YT-Indy cells than primary T cells. TCR-2A-RFP encoding lentiviral vector was functionally titered over YT-Indy cells by adding increasing amounts of virus to a fixed number of cells in a fixed volume. The resulting fluorescence was measured by flow cytometry 72 hours later. The proportions of positive cells in each condition were fit to a Poisson probability mass function by nonlinear least squares regression to determine a functional titer of 4.55 x 10^5^ infectious units/μL against YT-Indy (a). The same virus was also applied to an activated and proliferating culture of human T cells. In this experiment 150 μL of viral vector was added to 5x10^5^ T cells to yield a transduction e [file NIHPP2023.11.20.567960v1-supplement-1.pdf]

Suppl Fig 1

bioRxiv preprint doi: https://doi.org/10.1101/2023.11.20.567960; this version posted November 21, 2023. The copyright holder for this preprint (which was not certified by peer review) is the author/funder, who has granted bioRxiv a license to display the preprint in perpetuity. It is made available under aCC-BY 4.0 International license.

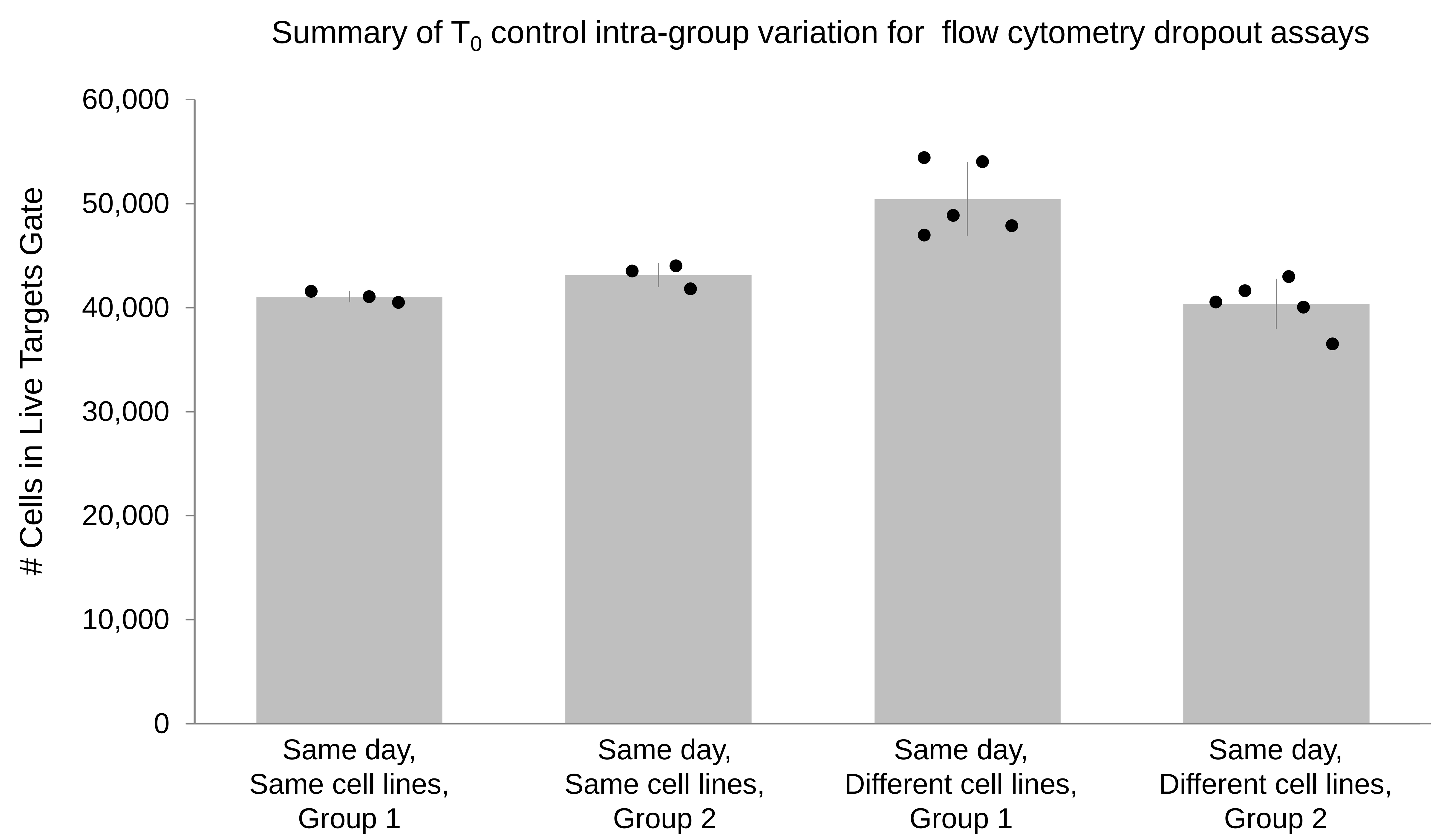

Suppl Fig 2

bioRxiv preprint doi: https://doi.org/10.1101/2023.11.20.567960; this version posted November 21, 2023. The copyright holder for this preprint (which was not certified by peer review) is the author/funder, who has granted bioRxiv a license to display the preprint in perpetuity. It is made available under aCC-BY 4.0 International license.

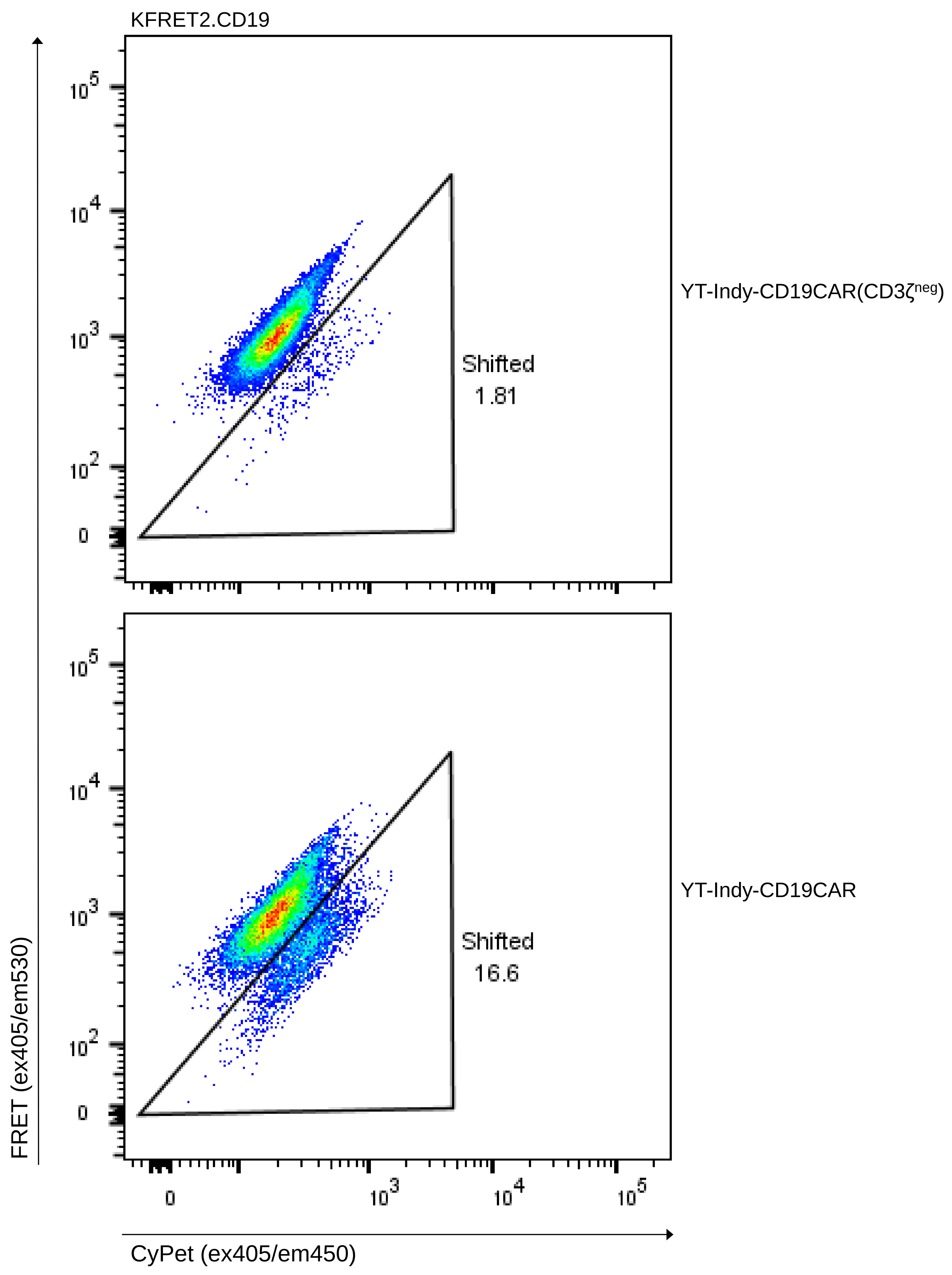

Suppl Fig 3

a

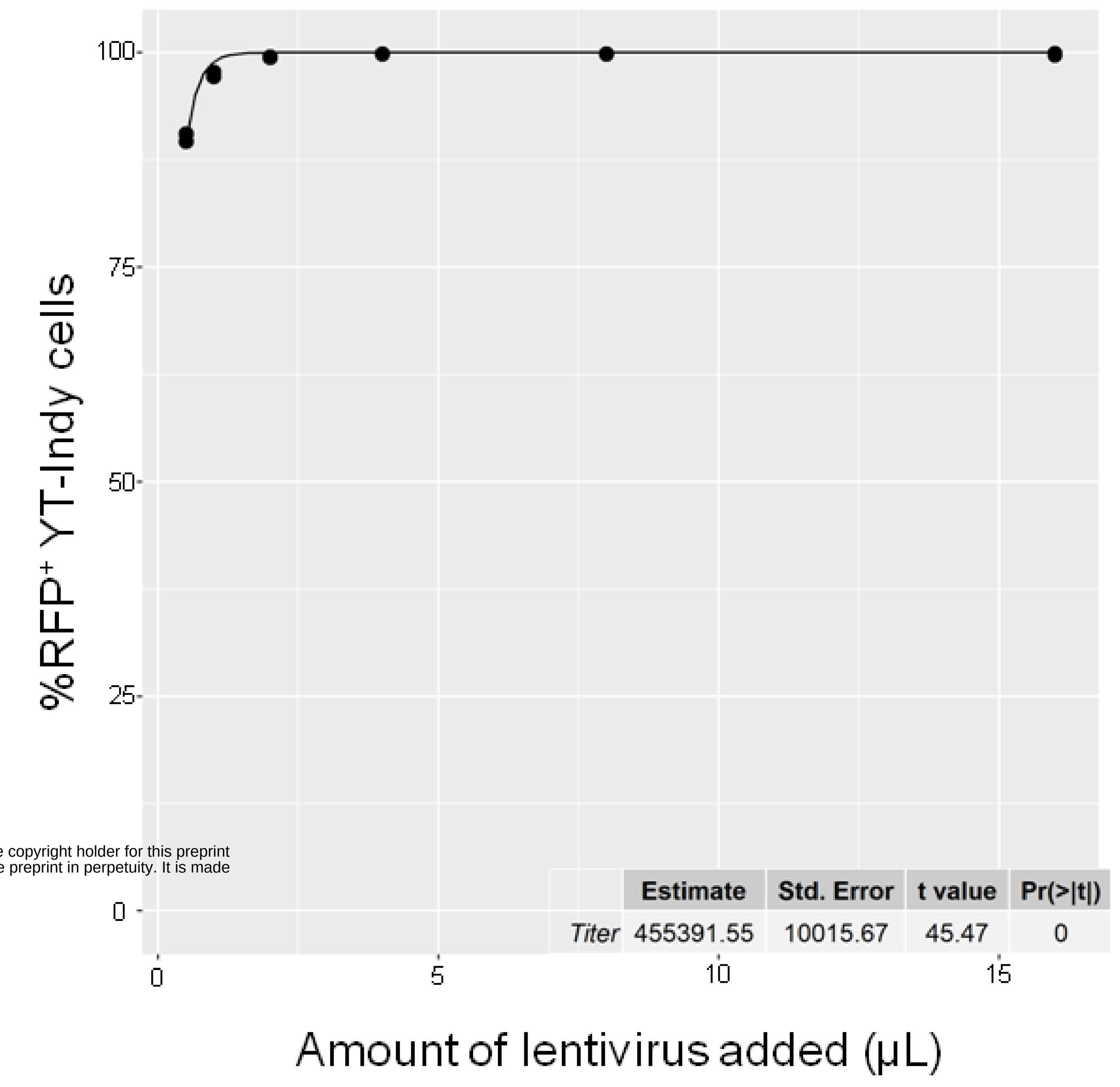

	Estimate	Std. Error	t value	Pr(>|t|)
Titer	455391.55	10015.67	45.47	0

b

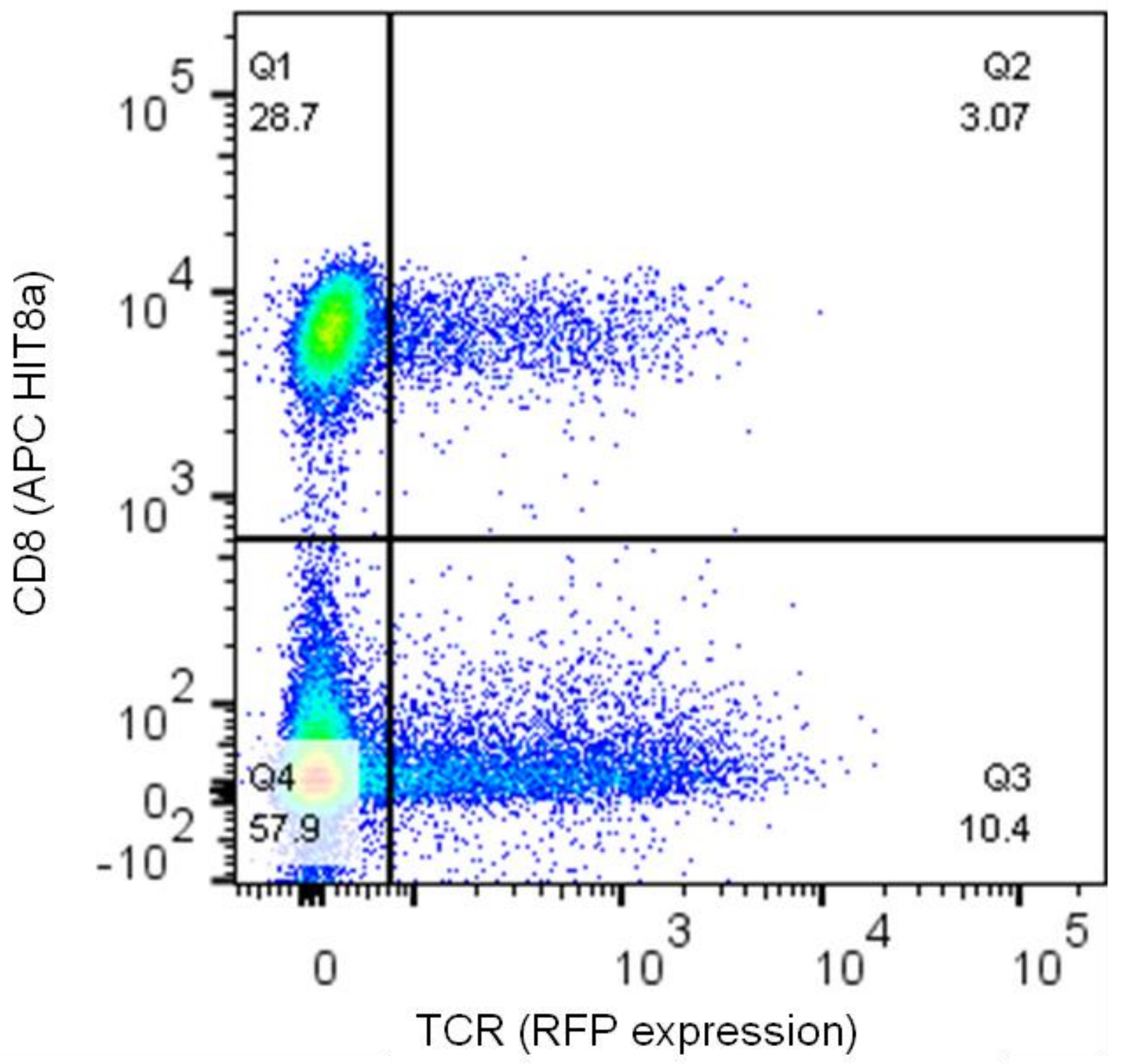

bioRxiv preprint doi: https://doi.org/10.1101/2023.11.20.567960; this version posted November 21, 2023. The copyright holder for this preprint (which was not certified by peer review) is the author/funder, who has granted bioRxiv a license to display the preprint in perpetuity. It is made available under aCC-BY 4.0 International license.

Suppl Fig 4

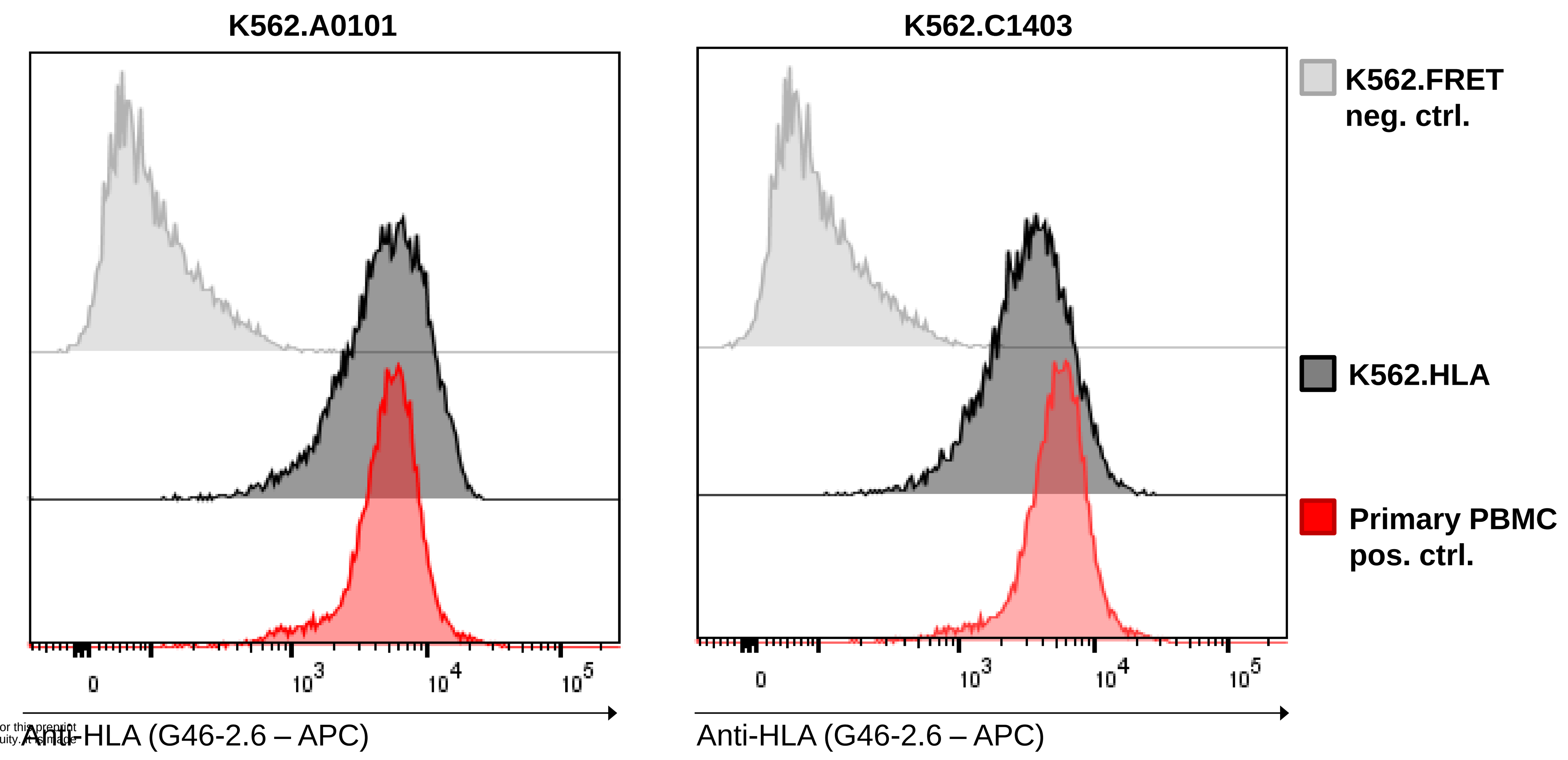

bioRxiv preprint doi: https://doi.org/10.1101/2023.11.20.567960; this version posted November 21, 2023. The copyright holder for this preprint (which was not certified by peer review) is the author/funder, who has granted bioRxiv a license to display the preprint in perpetuity. It is made available under aCC-BY 4.0 International license.

Suppl Fig 5

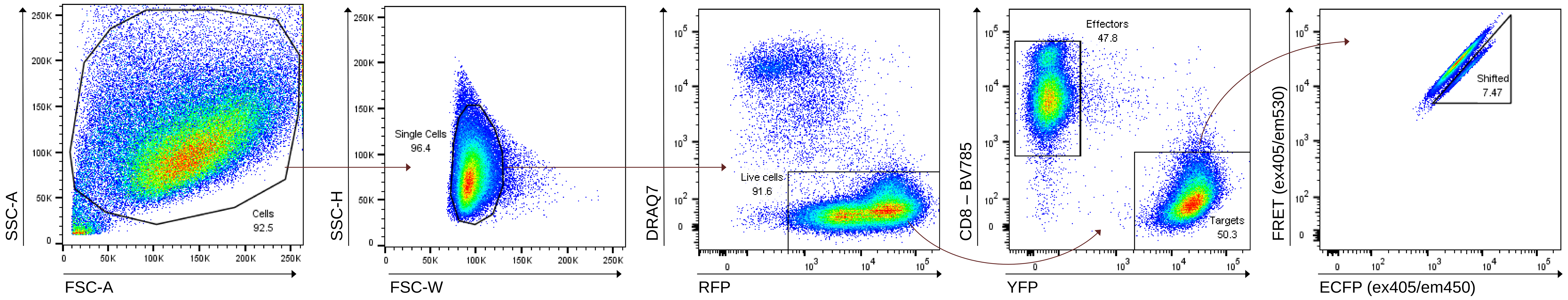

bioRxiv preprint doi: https://doi.org/10.1101/2023.11.20.567960; this version posted November 21, 2023. The copyright holder for this preprint (which was not certified by peer review) is the author/funder, who has granted bioRxiv a license to display the preprint in perpetuity. It is made available under aCC-BY 4.0 International license.

Suppl Fig 6

a

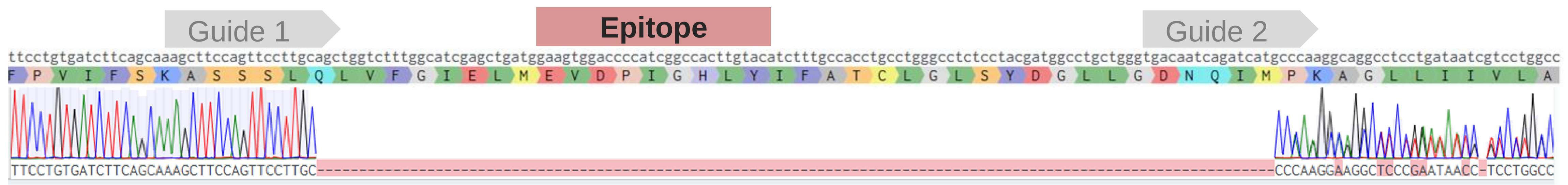

b

KFRET.A0101(MAGEA3^{ko}) – Clone 2B10
No minigene

KFRET.A0101(MAGEA3^{ko}) – Clone 2B10
+ Transduced MAGEA3$^{143\text{-}202}$ minigene

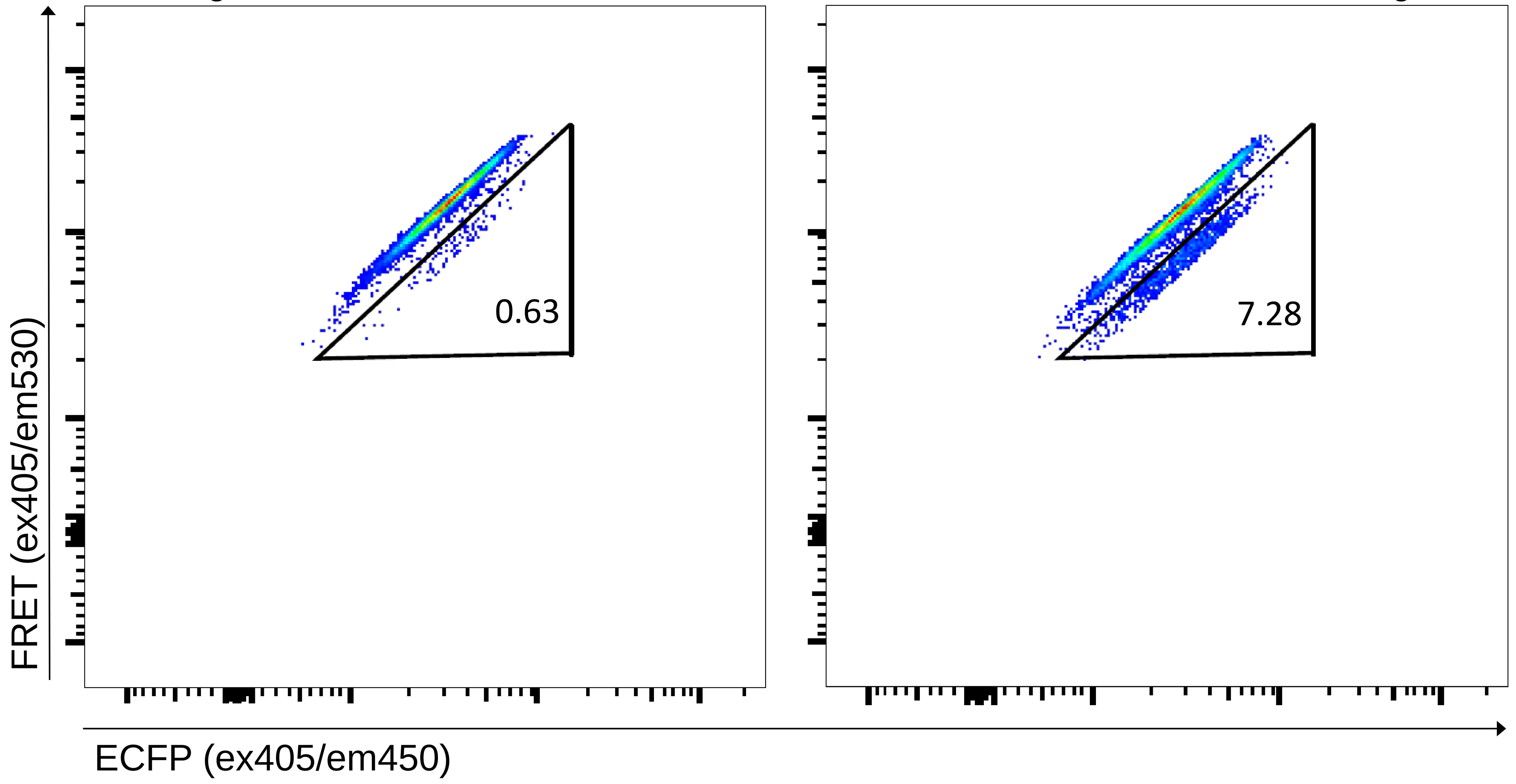

EB81.103 TCR-T

bioRxiv preprint doi: https://doi.org/10.1101/2023.11.20.567960; this version posted November 21, 2023. The copyright holder for this preprint (which was not certified by peer review) is the author/funder, who has granted bioRxiv a license to display the preprint in perpetuity. It is made available under aCC-BY 4.0 International license.

Suppl Fig 7

bioRxiv preprint doi: https://doi.org/10.1101/2023.11.20.567960; this version posted November 21, 2023. The copyright holder for this preprint (which was not certified by peer review) is the author/funder, who has granted bioRxiv a license to display the preprint in perpetuity. It is made available under aCC-BY 4.0 International license.

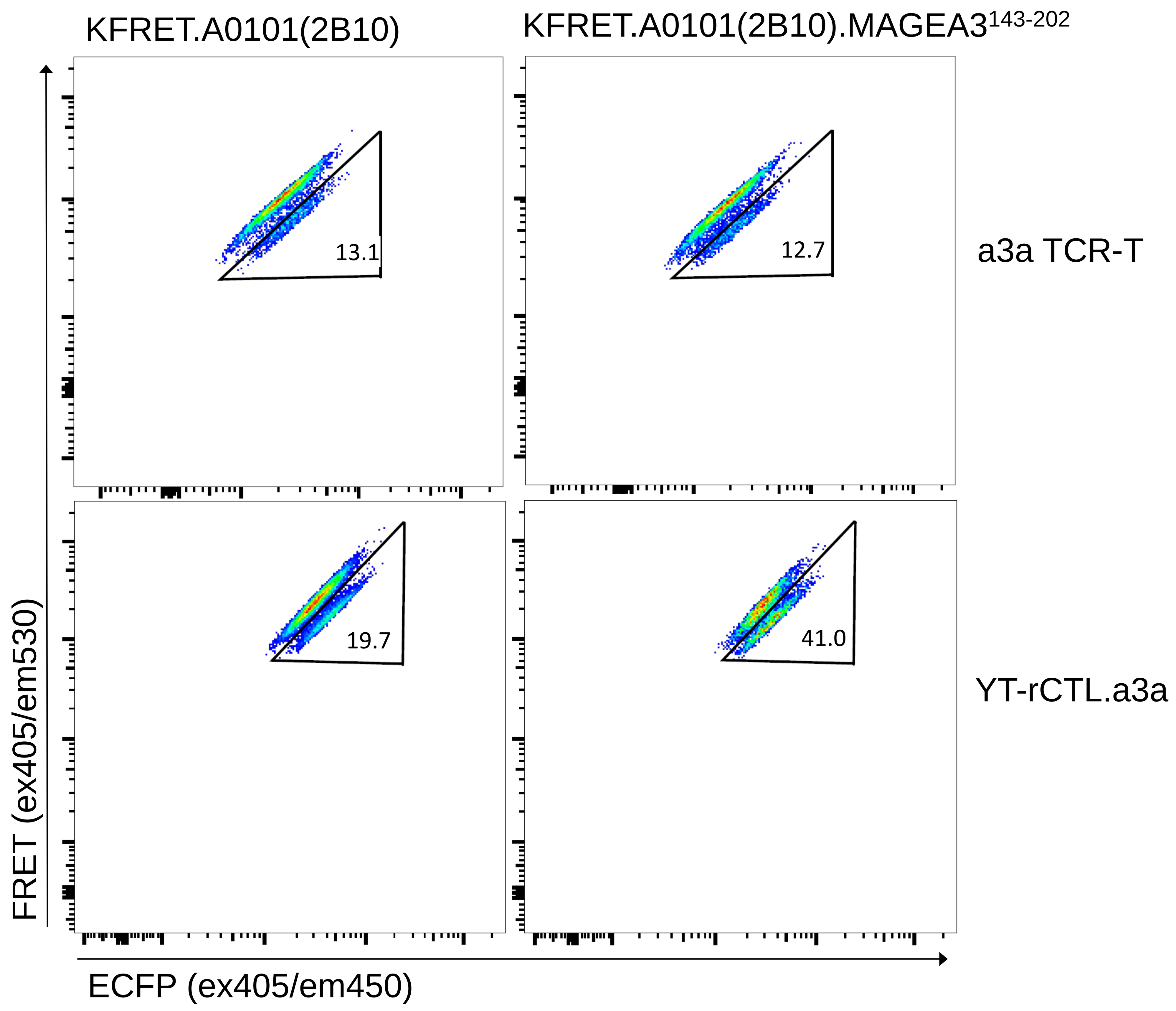

Suppl Fig 8

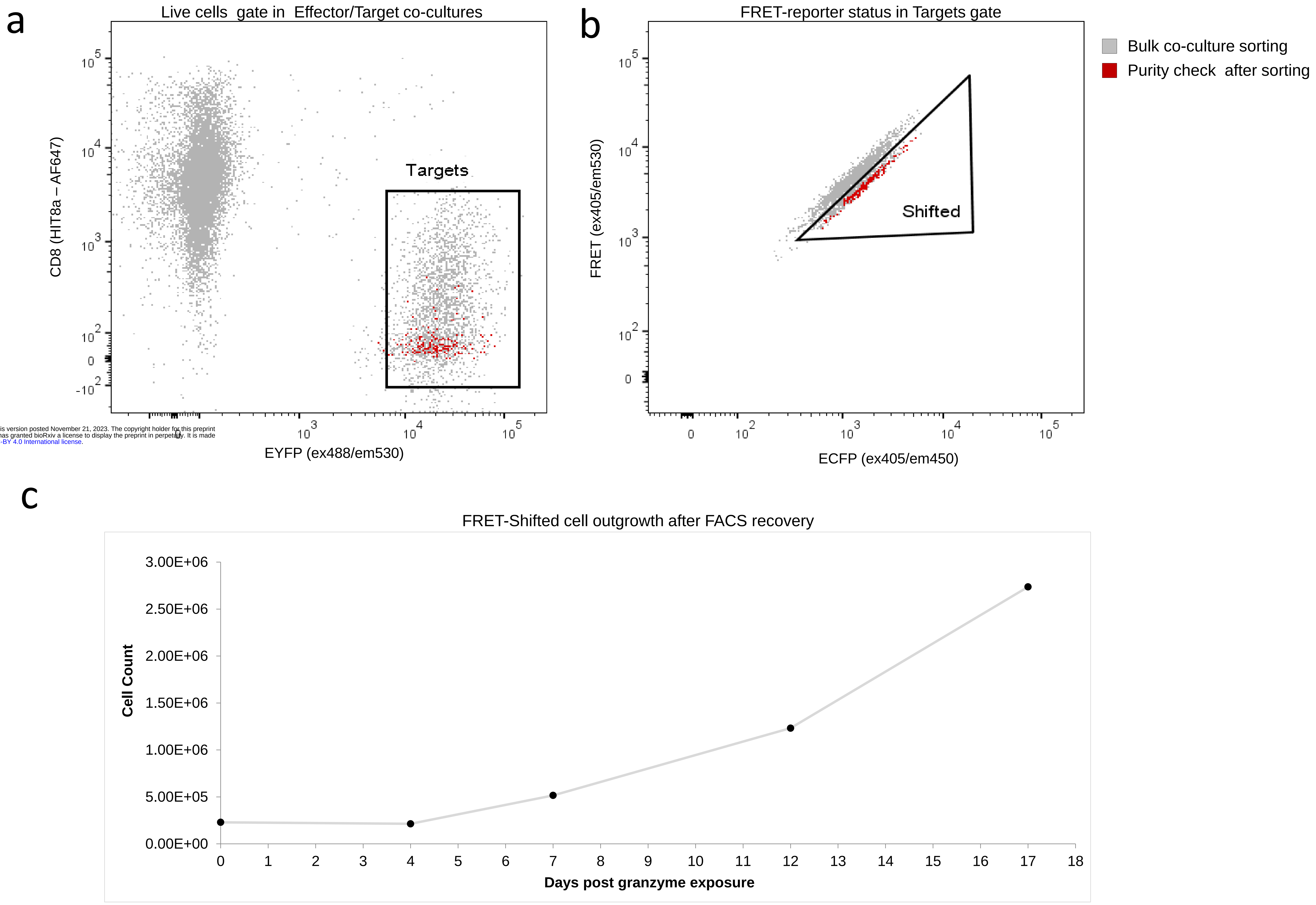

bioRxiv preprint doi: https://doi.org/10.1101/2023.11.20.567960; this version posted November 21, 2023. The copyright holder for this preprint (which was not certified by peer review) is the author/funder, who has granted bioRxiv a license to display the preprint in perpetuity. It is made available under aCC-BY 4.0 International license.

Suppl Fig 9

bioRxiv preprint doi: https://doi.org/10.1101/2023.11.20.567960; this version posted November 21, 2023. The copyright holder for this preprint (which was not certified by peer review) is the author/funder, who has granted bioRxiv a license to display the preprint in perpetuity. It is made available under aCC-BY 4.0 International license.

a

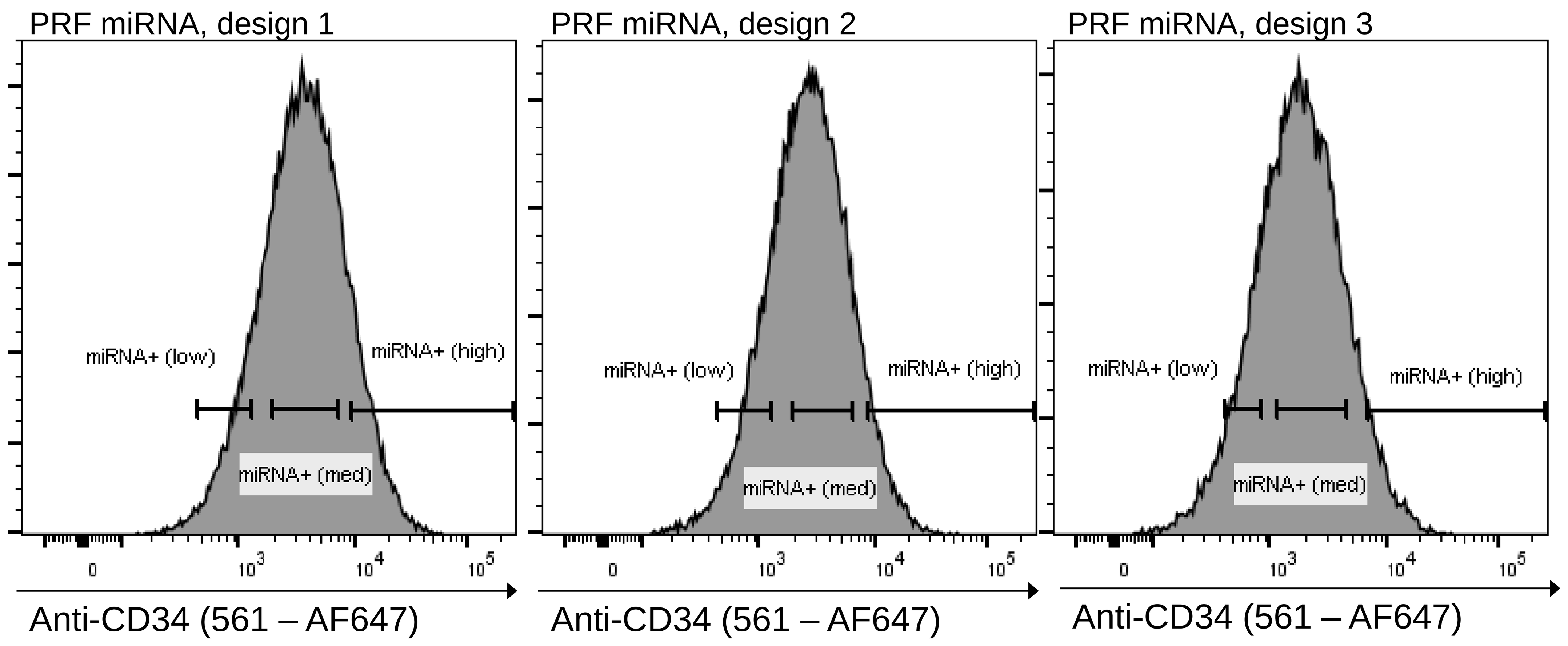

b

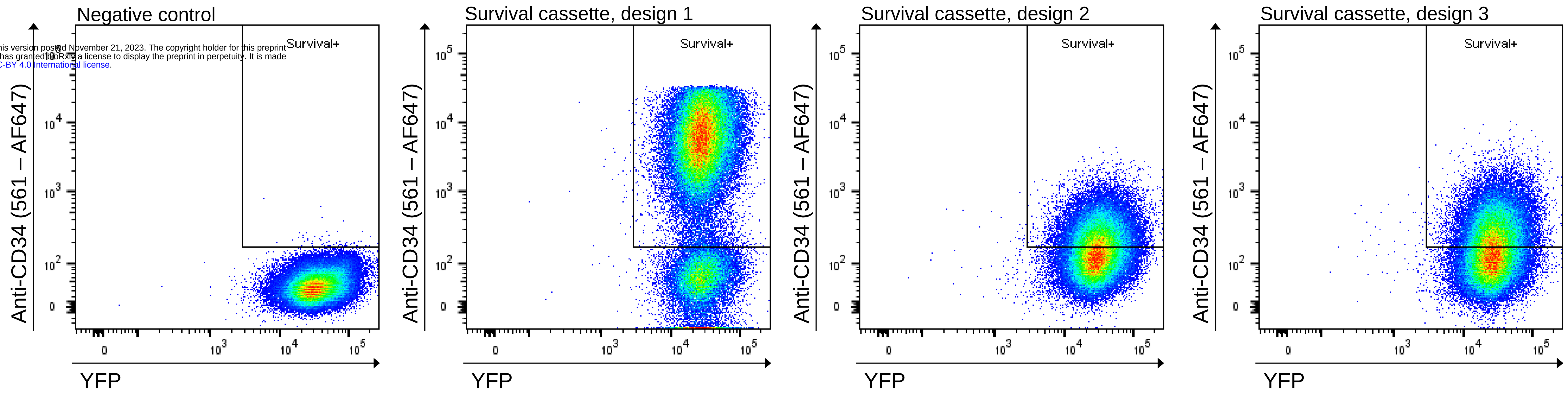

Suppl Fig 10

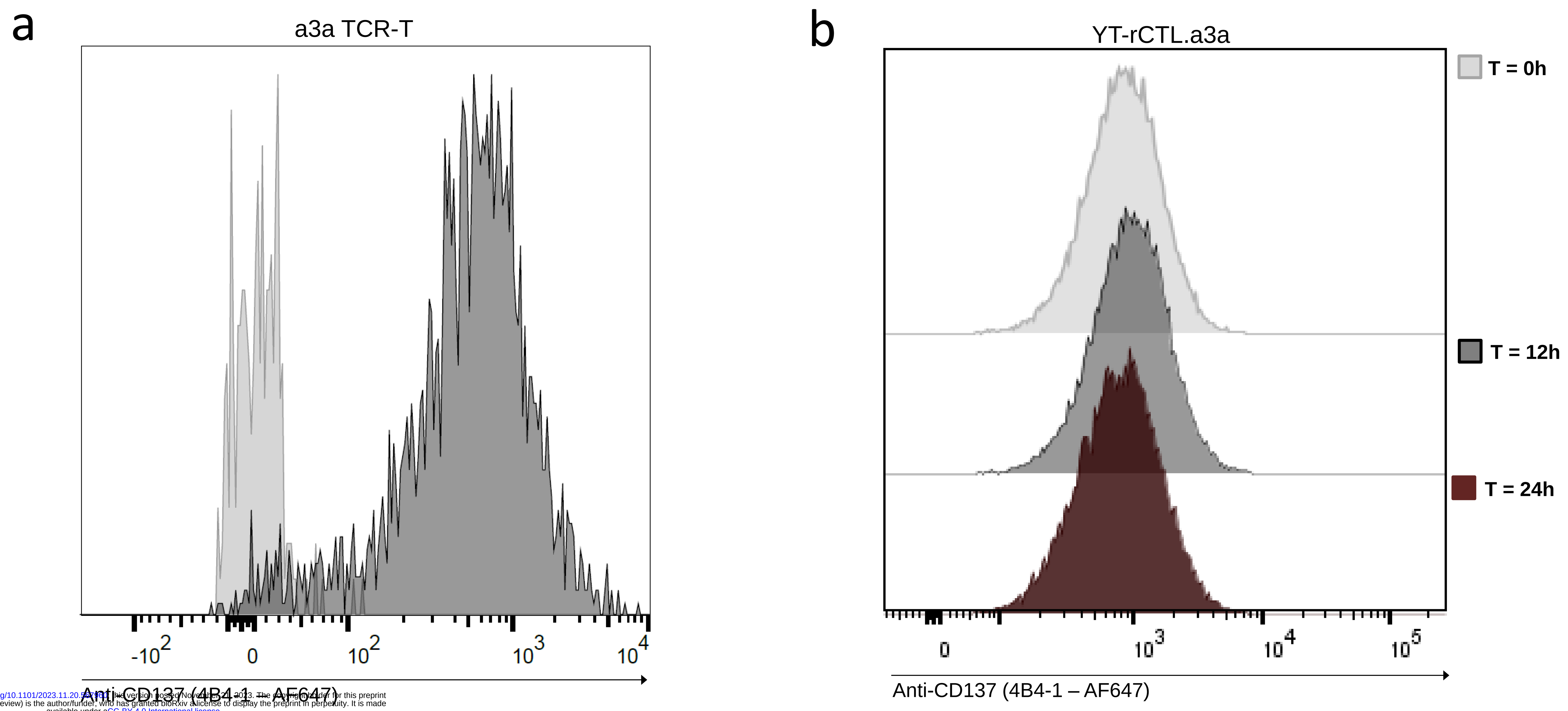

bioRxiv preprint doi: https://doi.org/10.1101/2023.11.20.567960; this version posted November 21, 2023. The copyright holder for this preprint (which was not certified by peer review) is the author/funder, who has granted bioRxiv a license to display the preprint in perpetuity. It is made available under aCC-BY 4.0 International license.
